# Cultural Identity and Internationally Adopted Children: Qualitative Approach to Parental Representations

**DOI:** 10.1371/journal.pone.0119635

**Published:** 2015-03-16

**Authors:** Aurélie Harf, Sara Skandrani, Jordan Sibeoni, Caroline Pontvert, Anne Revah-Levy, Marie Rose Moro

**Affiliations:** 1 Maison de Solenn-Maison des adolescents, Hôpital Cochin, AP-HP, Unité INSERM 669, Université Paris Descartes, Sorbonne Paris Cité, Paris, France; 2 EA4430, Université Paris Ouest Nanterre La Défense, Maison de Solenn-Maison des adolescents, Hôpital Cochin, AP-HP, Paris, France; 3 Centre de Soins Psychothérapeutiques de Transition pour Adolescents, Hôpital d’Argenteuil, Unité INSERM 669, Université Paris Descartes, Sorbonne Paris Cité, Paris, France; Beijing University of Posts and Telecommunications, CHINA

## Abstract

Approximately 30 000 children are adopted across national borders each year. A review of the literature on the cultural belonging of these internationally adopted children shows substantial differences between the literature from English-speaking countries and that from France and Europe in general. The objective of this study is to start from the discourse of French adoptive parents to explore their representations of their child's cultural belonging and their positions (their thoughts and representations) concerning connections with the child's country of birth and its culture. The study includes 51 French parents who adopted one or more children internationally. Each parent participated in a semi-structured interview, focused on the adoption procedure and their current associations with the child's birth country. The interviews were analyzed according to a qualitative phenomenological method, *Interpretative Phenomenological Analysis*. The principal themes that emerged from our analysis of the interviews made it possible to classify the parents into three different groups. The first group maintained no association with the child's country of birth and refused any multiplicity of cultural identities. The second group actively maintained regular associations with the child's country of birth and culture and affirmed that their family was multicultural. Finally, the third group adapted their associations with the child's birth country and its culture according to the child's questions and interests. Exploring parental representations of the adopted child enables professionals involved in adoption to provide better support to these families and to do preventive work at the level of family interactions.

## Introduction

International adoption refers to the legal adoption of children born in foreign countries. Worldwide, international adoption involves more than 30 000 children a year, moving between more than 100 countries [1]. France is one of the four countries receiving the largest number of internationally adopted children, after the United States, which is far ahead in first place, followed by Spain and Italy [1].

Internationally adopted children come from another country, another culture. They are unusual migrants for they have migrated alone. They belong to many places: their country of birth, place of early interactions, but also the receiving country, where they live—sometimes since they were very small. When children and their adoptive parents differ in their physical appearance, the children find themselves in what Lee calls the transracial adoption paradox, that is, they belong to the invisible majority in their family setting, but also belong to a visible minority through their different physical appearance [2].

Many situations may lead adoptive families to consider the question of the multiple cultures to which their children belong. These situations might be an experience of racism or discrimination, or questions that arose around a voyage to the child's country of birth, or at adolescence, a claim to belong to the country of birth. They might also arise from parental questions about the choice of a first name: should the first name be a French name? Should the first name given at birth be kept?

A review of the literature on the cultural belonging of internationally adopted children immediately shows a strong difference between the literature in Europe and in the United States. The majority position in Europe and especially in France does not consider the question of the country of birth and its culture. Instead, it stresses the feelings of belonging to the receiving country and its culture, for European authors argue that adopted children should have, above all, the same culture as their adoptive families. Promoting connections with the birth culture creates the risk of accentuating the differences between the child and the parents, differences that should be erased to construct a family. What the success of the adoption and of the child’s development depend on is the child’s inscription in the intergenerational history of the adoptive parents [3,4,5].

A different position, dominant in the English-language literature, insists on the contrary on the importance of maintaining connections with the country of birth and its culture. On the model of studies of immigrants, numerous authors have studied the issue of the cultural belonging and identity of adopted children. Cultural identity is defined as the entire set of beliefs, social behaviors, rites, customs, traditions, values, language, and institutions of a given culture [6,7,8].

The cultural competence of adoptees in their culture of birth is developed through their participation in cultural activities: learning the language, participating in holidays, in meals where the traditional food of the country of birth is served, developing awareness of traditions, listening to music and seeing films from that country, and becoming conscious of one's physical resemblance to people of the same ethnic and cultural group [8]. This cultural socialization is thought to promote children's pride in their cultural heritage and to enable them both to prepare to live as a member of an ethnic minority and to learn strategies to cope with racism and discrimination [2].

These studies continue a line of studies about immigrants and their children that have demonstrated that the development of a strong cultural identity is key to their well-being and psychological adaptation. It also protects against the development of psychopathologic symptoms, in particular, depression and attempted suicide [9,10].

Nonetheless, the results of studies seeking to show a correlation between a strong and positive cultural identity and better psychological development of internationally adopted children are not uniformly consistent. Some studies show a correlation between self-esteem or psychological well-being and the level of cultural identity [11,12,13]. Others report a correlation between bicultural socialization and such positive psychological outcomes as higher self-esteem, higher educational achievement, and higher levels of adult adjustment [2,14,15], although other studies have found no such correlation [16,17]. A literature review confirmed that the relation between cultural and ethnic identity development and psychological adjustment in adopted children is complicated: some studies have found that ethnic and cultural identity can play an important role in the promotion of self-esteem and positive adjustment, while others conclude that the relation is less significant [18].

Many studies have examined the influence of parents' attitudes on the development of identity in adopted children, especially in multicultural families. Several show that the children's level of bicultural competence depends on the parents' beliefs about the importance of bicultural socialization: how much do they want their child to participate in cultural events of his or her birth country, to learn its history and its language? Does the parents' social network include people who come from the same country or belong to the same ethnic group as the child who can serve as role models? Have the parents taken active steps to connect the child with the community from the birth country and to encourage identification with the ethnic group, and the claiming of the child’s cultural heritage? [19,20,21,6,7,8,22,23,24,12,25]

There is thus an abundant English-language literature in the field of adoption around the question of the cultural identity of adopted children, and it recommends that parents maintain connections with the birth culture of their child. These studies have generally not used qualitative methods to explore the adoptive parents’ point of view about their child's cultural belonging. Some have explored parents' feelings on this topic [26] and revealed the contradictions that these families experience in their identity-work [27,28,29]. Others have focused on communication within adoptive families, as an aspect of family interactions [30,31,32]. Nonetheless, qualitative studies are recommended when the goal is to uncover the common and unique experiences of individuals who have firsthand knowledge of the phenomenon of interest [33,34].

In view of the differences observed in the literature between Europe and the United States and because little is known about the parental representations of the cultural belonging of adopted children in a French context, we approached this research as a qualitative question. We think it essential to begin our analysis with the discourse of adoptive parents, to see how they do or do not appropriate different theoretical positions about their children’s cultural identity. Our objective is thus to explore qualitatively adoptive parents' representations of their child's cultural belonging in a French setting. The study presented here examines the ways that parents describe their child's cultural identity. Our qualitative methodology gives us access to the arguments parents use to explain their beliefs: the reasons why they consider it important to promote connections with the child's country of birth and its culture, or inversely, why they think that it is not beneficial for the child.

We further note that the differences between countries in the treatment of adoptees' cultural belonging make the use of qualitative methods especially relevant. The importance or even relevance of the culture of origin differs between countries, according to sociocultural context [35,36]. In our study, qualitative methods enable us to explore how French adoptive parents relate to the positions described in both the European and the English-language literature. We aim to identify the different parental strategies employed from what parents said in our interviews about their experiences and about the meaning they attribute to them.

This work follows and is related to an initial study that explored the experience of adoptive parents at the moment of the first parent-child encounters in international adoptions [37]. We anticipate that it will contribute to setting up methods of early prevention through work with adoptive families on family interaction. The issue of the adopted children's cultural belongings can become a point of distress during the construction of their identity and within the parent-child relationship [38,11,39].

## Method

### Participants

Eligible participants adopted at least one child from a country other than France. The sample comprised 51 French adoptive parents who volunteered to participate in the study: 13 fathers and 38 mothers. When children had been adopted by couples, we asked both parents to participate in the research. Interviews were performed separately for each parent. The 51 parents in this study included 12 couples in which both parents (father and mother) participated, that is, 24 parents. Of the remaining 27 parents, 26 were mothers: 16 adopted as single mothers, while 10 adopted with their husbands, who did not participate in the study. The final parent included is a father, who is married but whose wife did not participate.

Eleven parents had two internationally adopted children and were each interviewed twice (one interview for each child). Two parents had three internationally adopted children and were each interviewed three times. We thus had a total of 66 interviews. The interviews took place between May 2011 and January 2013.

Parents’ ages at the time of their children’s adoptions ranged from 28 to 49 years (mean age = 41) and at the time of the interview from 31 to 60 years (mean age = 47). Parents lived in urban areas of France. Most (86%) were college-educated professionals.

**Fig 1:**
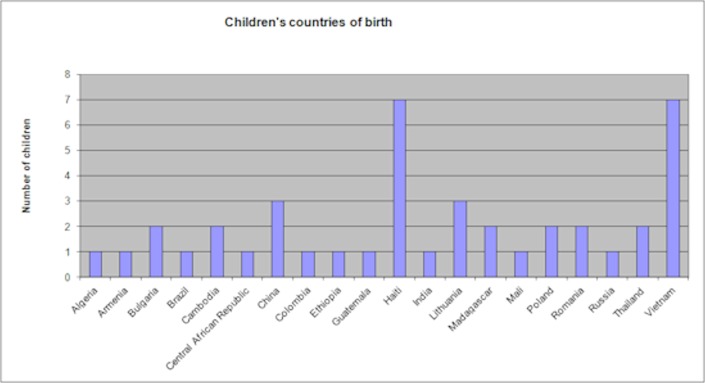
Children’s countries of birth. Fig. 1 describes the countries of birth of the children adopted by parents included in this study. One child was adopted in Algeria, 1 in Armenia, 2 in Bulgaria, 1 in Brazil, 2 in Cambodia, 1 in Central African Republic, 3 in China, 1 in Colombia, 1 in Ethiopia, 1 in Guatemala, 7 in Haiti, 1 in India, 3 in Lithuania, 2 in Madagascar, 1 in Mali, 2 in Poland, 2 in Romania, 1 in Russia, 2 in Thailand, and 7 in Vietnam.

Of the 48 internationally adopted children, 27 were girls and 21 boys. At the time of adoption, their ages ranged from 2 weeks to 7 years (mean age = 2.1 years). Eighteen children were younger than 1 year at the moment of their adoption, 13 were 1 or 2 years old, and 17 were 3 years or older. Fig. 1 presents the children’s countries of birth.

At the time of the interview, the children’s ages ranged from 15 months to 17 years (mean age = 8.6 years). The time between the adoption and the interview ranged from 1 year to 16 years (mean = 6.6). Of the 66 interviews, 15 concerned children adopted within two years of the interview, 11, 2 to 5 years before the interview, and 40, children adopted more than 5 years earlier. Fourteen parents had both adopted and biological children. The number of children per family ranged from 1 to 4, and the number of adopted children from 1 to 3.

An effort was made to vary the sample in terms of age, life stage (i.e., families with young children as well as those with adolescents), and family structure (single parents and married couples). Sampling in qualitative research involves purposive sampling of individuals liable to provide the most informative description of the phenomenon under study [40]. Our sampling technique was indeed purposive, because we selected subjects who were typical of the population of interest [41]. The participants were recruited in the general population through adoption associations and connections between adoptive parents.

### Data Collection Procedure

Data were collected via semi-structured interviews. The authors reviewed the international adoption literature to develop a guide for these parental interviews. The broad topics covered included the steps in the adoption process, the choice of country, the trip to the child's native country, what is known of the child’s pre-adoption history, links to the child’s country of birth and its culture, and any racism or discrimination the child might have experienced. The interview protocol that guided this research is included in the appendix (S1 Appendix).

Questions were designed to obtain specific information while remaining flexible so that the interviewees could tell their stories. Open-ended questions allowed participants to interpret the meaning of the question and respond according to their personal feelings. Interviewers used prompts and probes as needed to enrich the discussion. We chose to collect data through semi-structured interviews because this method combines an approximate standardization of questions with the opportunity for subjects and interviewers to expand their answers when appropriate. The interviewing process produced data that were both deep and broad and focused on the research question: the connections that adoptive parents maintained with their child's country of birth and its culture and the parents' representations of their child's cultural identity.

Parents chose the interview site: the researcher's office or their own home. The participants and what they had to say determined the length of the interviews, which averaged one hour. Every interview was audio-taped for later transcription, with the participants’ permission, and transcribed verbatim in French. Two different researchers conducted these interviews, separately. Each had training in the fields of adoption and qualitative research methods (AH, SS).

### Data analysis

A phenomenological research design was employed to understand the parents’ representations of their children’s cultural identity. Phenomenology is a nonprescriptive approach to research that allows the essence of experience to emerge, yet anchors data analysis in the participants’ unique representations [41]. The aim is to explore personal experience and the subjective perception of an object or event. Our research approach is phenomenological in that it involves detailed examination of the participants’ personal perceptions and lived experiences.

To analyze our interview data, we used the *Interpretative Phenomenological Analysis (IPA)* method [42,43], an established qualitative methodology used to explore in depth how individuals perceive particular situations they are facing and how they make sense of their personal and social world. Following the IPA, we conducted an in-depth qualitative analysis. It began with a detailed case-by-case study of each interview transcript, according to an iterative inductive process. We started with several close detailed readings of each interview to provide a holistic perspective, noting points of interest and significance. Through a step-by-step analysis, we proceeded to the description of analytic themes and their interconnections, while taking care to preserve a link back to the original account. IPA thus involves navigating between different levels of interpretation [42]. The last stage involves the production of a coherent ordered table of the themes [43]. The procedure for data analysis was inductive, as the analysis of data from the literature was performed secondarily. The size of the sample was determined by data saturation, that is, the point at which in-depth analysis of the interviews no longer results in the emergence of new themes.

Because of the qualitative methods chosen (theoretical sampling and no statistics), we had no preconceived ideas of the number of parents to include. The data collection and analysis took place simultaneously, and inclusions continued for as long as the analysis of the material continued to provide new information useful for the exploration of the phenomenon. In this type of study, sampling, continued data collection, and the development of themes and subthemes are interdependent. When data analysis becomes redundant and no longer provides new elements, it is time to conclude that data saturation has been reached and to stop including new subjects. As with the inductive procedure, data collection and analysis of results continued simultaneously until sample saturation. The analysis influences the data collection by leading us to redefine the research question, to find counter-examples, and to investigate new pathways [44].

Throughout, we employed computer software to assist our analysis: QSR NVivo9 for data management, topic extraction from the transcripts, and their thematic recoding.

### Validity

To insure the validity of our qualitative research, we compared the researchers’ coding. Two trained researchers (AH, SS) independently coded and interpreted all of the parent interview data. The two coders discussed the emerging codes in repeated meetings with other members of the research team (ARL, MRM, JS and CP) who had read the transcripts. These discussions helped to identify potential themes in the data that might not yet have been captured by the codes and enabled us to clarify or modify the coding to increase the consistency and coherence of the analysis by ensuring that the themes we identified accurately reflected the data and that the analysis was not confined to one perspective. Multiple discussions allowed us to eliminate systematic differences due to variations in interpretation. Validity was also enhanced by the care we took to distinguish clearly between what respondents said and how we interpreted it or took account of it [43].

Member-checking (also known as informant feedback or respondent validation) was practiced, for it is a vital way for interpretive researchers to verify the trustworthiness of their research [45]. When the qualitative analysis of the parents’ data was completed, a summary of the thematic results was mailed to the parents, who were asked to provide feedback, reactions, and comments. Participants were also asked to share these preliminary results with spouses who had been unable to take part in the interview. Ten of the 51 parents provided written or verbal feedback, which was incorporated in the final results. This methodological aspect of the study provided a source of testimonial validity for the qualitative results; it also enabled us to take participant feedback into account in their interpretation [46] and to assess the degree to which the themes resonated with the parents’ experience.

### Ethics statement

Parents were fully informed of the voluntary nature and the goals of the study. Written informed consent was obtained from all parents included in the study before the interview. Participants were informed that all responses would be confidential, that the transcripts would have no identifying information, and that they would be free to withdraw at any time. All identifying information was removed from the transcripts, and participant anonymity further ensured by disguising or withholding of descriptive data. The Ethical Review Committee (Institutional Review Board of Paris North Hospitals, Paris 7 University, AP-HP, N° IRB00006477) approved this research protocol.

## Results

The phenomenological analysis of the 51 parents’ interviews showed three groups of parents, corresponding to three types of parental representations of their adopted child and his or her cultural belonging:

- GROUP 1: The parents maintained no association with the children's country of birth. They refused any multiplicity of cultural identities.
- GROUP 2: The parents actively maintained regular associations with the children's country of birth and culture and affirmed that they were a multicultural family.
- GROUP 3: Parents adapted their links to the country of birth and culture according to the child's questions and interests. They accepted the multiplicity of their child's feelings of cultural belonging.

The results below include excerpts of respondents’ verbatim accounts, used to exemplify the recurrent underlying themes. To protect confidentiality, identifying information has been removed from the quotations presented. The verbatim account has been freely translated into English for the sole purpose of this article; the main objective of the translation is to preserve the essential meaning, content, and insofar as possible general tone. Ellipses within brackets indicate the deletion of a part of the sentence. For greater clarity and to preserve the parents' anonymity, parents are designated as Mothers 1 to 38 and Fathers 1 to 13. The child's age at the time of the interview has been added to the end of each quotation, following the parent's number (e.g., Mother 5, of a 6-year-old).

Table 1 summarizes the themes identified and their characteristics for the three groups. Table 2 presents the children’s age at adoption according to group, Table 3, children’s age at time of the study according to group and Table 4, time between the adoption and the interview according to group.

**Table 1:** Themes found in the three groups.

	GROUP 112 parents	GROUP 218 parents	GROUP 321 parents
**Choice of country**	ChanceorMinimize the difference in the child's and parents' physical appearance	Attraction to the countryand/orSeeking a visible difference between child and parents	Compromise between affective factors and practical constraints
**Experience of racism**	Denial or trivialization	Talk to the child about racism	Talk about racismTeach self-defense strategies against racism
**Child's cultural belonging**	Refusal of cultural belonging other than French	Actively defend the child's bicultural identity	Promote links with the country of birth and its culture, when the child so requests
**Child's history before adoption**	No research	No research	Active research
**Contacts with adoptive parents**	No contacts	Adoptive-parent associations, internet groups	Support from adoptive-parent groups
**Travel to the country of birth**	No plan for a trip	Trip already made or being planned	Trip if child asks for it

**Table 2:** Children's age at adoption (according to group).

AGE AT ADOPTION	GROUP 1	GROUP 2	GROUP 3	
< 1 year	5	11	6	21
Aged 1 or 2 years	6	6	11	23
> 2 years	4	7	10	21
	15	24	27	66

**Table 3:** Children's age at time of the study (according to group).

AGE AT TIME OF THE STUDY	GROUP 1	GROUP 2	GROUP 3	
< 12 years	10	16	19	44
≥ 12 years	5	8	8	21
	15	24	27	66

**Table 4:** Time between the adoption and the interview (according to group).

TIME BETWEEN THE ADOPTION AND THE INTERVIEW	GROUP 1	GROUP 2	GROUP 3	
< 3 years	6	6	8	20
Between 3 and 7 years	2	8	13	23
> 7 years	7	10	6	23
	15	24	27	66

- Group 1: Absence of associations with the child's country of birth, refusal of any multiplicity of cultural identities for their children

## 12 parents (15 interviews): 24%

The parents in group 1 reported that they have no association with the child's country of birth in their daily lives and no interest in this country.

### Choice of country

Seven parents in group 1 stated that they had chosen the country by chance or by expedience.

*“We went to China*. *But it was chance*. *In any case*, *we wanted to adopt*, *so it didn't matter what country*.” (Father 5, of a 4-year-old)

The other 5 parents of group 1 explained that they had chosen the country so that there would not be too great a difference in physical appearance.

*“I said to myself*, *a black child*, *it will be obvious that I adopted*.” (Mother 15, of a 10-year-old)

*“We went to Romania*, *because*, *let's put it this way*, *the children were more or less European*.” (Father 1, of an 11-year-old)

### Experience of racism and discrimination

Parents in group 1 did not talk about racism with their child and considered that it was not really a problem.

*“Children tease; but without any more*, *it's just teasing (…); he's too young to have faced this*, *I think*.” (Father 6, of a 5-year-old)

### Child’s cultural belonging

The parents in group 1 stressed that their children are French and that their culture is only the culture of the country of adoption, that is, French culture. The culture of the child's country of birth did not interest them. For them, the child's integration requires refusal of any association with the culture of the country of birth.

*“[My daughter]*, *for me*, *she's French*, *now her country is France*, *she will have lived in France*, *not in Mali (…) Her cultural origins*, *they will be ours*, *I think*.” (Mother 9, of a 4-year-old)

### Child's history before adoption

The parents in group 1 made no active effort to learn about elements of the child's history before adoption.

*“You have to let go of the past*.” (Father 1, of an 11-year-old)

### Contacts with other adoptive parents

Finally, parents in group 1 did not want to maintain contact with other adoptive parents because they perceived that as a stigma. They insisted that they are parents like any others.

*“The meetings of adopters*, *for example*, *I don't like that because*, *it's a little like a ghetto*.” (Mother 9, of a 4-year-old)

### Travel to the country of birth

The parents in group 1 stated that they did not have plans to return to the child's country of birth, now or later.

*“I don't have any desire to go back there*. *Finally*, *why not*, *but there are so many other things to see*!” (Father 5, of a 4-year-old)

- Group 2: Regular associations with the child's country of birth and its culture. Affirmation of a multicultural family

## 18 parents (24 interviews): 35%

The parents in group 2 reported multiple and frequent associations with their child's country of birth and its culture. They follow the news in that country closely. They have contacts with people living there and with people from there living in France. These parents stressed the links that they themselves have with the child's country of birth and its culture, as though the adoption had brought them even closer to it. They described it as their second country, after France.

*“I have a very strong link to Haiti*. *If I could*, *I would go there every year (…) We can talk about Haiti without it being*, *Haiti*, *it's your thing*, *before me*. *Haiti*, *it's also something that belongs to both of us*.” (Mother 19, of a 9-year-old)

*“It’s sort of our family ambience*, *our way of living*, *we were after all immersed in Asia even before the adoption*.” (Mother 24, of a 7-year-old)

*“I'm learning Chinese*. *I decided*, *so I started that*, *last week*, *but it's not* … *it's nothing to do with my son*, *it's me*: *I've had a great desire to learn Mandarin*.” (Mother 3, of an 8-year-old)

### Choice of country

Eight parents in group 2 had already visited the country, at least once and sometimes several times, before deciding to adopt there. In all the interviews in group 2, the parents said they were attracted by the country or the region.

*“I love Asia a lot*, *I traveled a lot in Asia a long time ago; I had also been to Vietnam a very long time ago*, *I loved it*.” (Mother 24, of a 7-year-old)

The choice of country can also be associated with the question of skin color. Six parents proclaimed that they were a family with visible differences.

*“I had a little preference for black skin (…) We are not the same color*, *I think that pleases us; anyway*, *I like it*. *Perhaps I wanted a child who did not look like me*.” (Mother 30, of a 6-year-old)

### Experience of racism and discrimination

The responses to the racist comments that the child might face were variable. Eight parents' responses concerned details about the country or its culture. Ten other parents in group 2 explained to the child what discrimination is.

*“I think that we are all children of the world*, *and what is important is to say that we respect all human beings similarly*, *regardless of their origins*.” (Father 7, of an 8-year-old)

### Child’s cultural belonging

All the parents in group 2 actively defended their child's bicultural identity (culture of the country of birth and of the country of adoption). To achieve this goal, they actively supported the child’s links with the culture of the country of birth, through its language, its food, its holidays, traditions, etc.

*“I think we must keep his origins for him*, *his personality*, *his culture*.” (Mother 6, of a 13-year-old)

*“What I tell her is that she's lucky to have two cultures and she should take the best in each of them*.”(Mother 11, of an 8-year-old)

### Child's history before adoption

All of the parents in group 2 indicated that they give their children all the information they have about their history and birth parents but do not actively search for information about the birth family.

*“So my son lived in a slum*. *His mother sold bread*. *Uh*, *and what I know is that she could not always stay with him and would sometimes leave him with his 6-year-old sister*. *So*, *uh*, *that's what I know about her*, *her past*, *his daily life; I don't know more but*, *well*, *there aren't any explanations*.” (Mother 11, of a 8-year-old)

### Contacts with other adoptive parents

All the parents in group 2 are in regular contact with other adoptive parents. They are very active in internet forums or in associations of adoptive parents. Some take an active role in helping future adoptive parents or in setting up humanitarian aid.

*“I'm the president of the SOS Haiti adopted children’s group*. *It was set up after the earthquake on January 12*. *(…) And uh after that first commitment*, *I took on another one; I set up an association to help women*. *I organized an auction to raise funds for Haiti*, *with other adoptive mothers*.” (Mother 30, of a 6-year-old)

### Travel to the country of birth

Nine parents had already returned with the child to the country of birth, sometimes for humanitarian reasons. The other 9 parents planned to travel there with the child.

*“I've already promised [him] that when he's 8 or 10 years old*, *we will go to his country*.” (Mother 1, of a 4-year-old)

- Group 3: Links with the country of birth and its culture depend on the child's questions and interest. Acceptance of the multiplicity of the child's feelings of cultural belonging

## 21 parents (27 interviews): 41%

Parents in group 3 had few associations with the child's country of birth and its culture in their daily lives. Nonetheless when the children asked questions about their lives before the adoption, their birth parents, their skin color, or experiences of racism, the parents in this group talked to the child about the country of birth and its culture. They stated that they would provide support should the child choose to draw closer to that culture.

### Choice of country

The choice of country for the parents in group 3 was essentially a compromise between emotional aspects and constraints.

*“Since I'm a single mother*, *that limits the number of countries enormously*, *and then*, *just then*, *I met a woman*, *by chance*, *who had adopted a little boy in Haiti*. *And the meeting with her and the child was so sweet*.” (Mother 33, of a 10-year-old)

### Experience of racism and discrimination

The parents in group 3 recognized the consequences in the child's daily life of the difference in skin color and the visibility of the adoption. Nineteen parents said that they think it is necessary to talk with their children about racism and that there is a risk that they will experience racism and discrimination. Eleven stressed the importance of teaching children how to defend themselves when they do experience it.

*“Is [he] going to suffer from something in this country that is so racist*? *Yes*, *and I am trying to raise him with the weapons to defend himself*.” (Mother 2, of a 2-year-old)

Twelve parents underlined that experiences of racism are the occasion to talk about the differences between human beings.

*“I have already told her that sometimes perhaps there will be people who don't like anyone different from themselves*, *who might make nasty comments*, *but it's just that they don't know better*, *or they are afraid of what is different*.” (Mother 16, of a 9-year-old)

Six parents reported that in response to the child's experiences of discrimination, they talk about the value and strong points of the culture of the country of birth. They consider that the best weapon against racism is increasing the child's self-esteem.

*“I talk about the really positive aspects*, *such as the culture*, *musicians*, *writers*, *and poets* …” (Mother 23, of a 7-year-old)

*“It really matters to me that she be proud of the country she comes from*.” (Father 11, of a 5-year-old)

#### Child’s cultural belonging

Parents in group 3 reported that they accept their children's feelings of belonging to their country of birth because they consider it is part of their history. Nonetheless, 14 parents also insisted on their being anchored as well in the adoptive country's culture, because they think that children have above all a need to feel that they belong to the culture they live in.

*“She is being raised by a mother whose culture is French*, *she goes to a French school*, *her cultural background is French*. *That's it*. *And at the same time it is true that I think that nonetheless*, *it [Haitian culture] is a culture that probably has a special meaning for her*.” (Mother 21, of a 12-year-old)

The parents in this group consider the child's age and pace of development important as well: they promote links with the country of birth and its culture at the child's own pace, that is, when the child wants to develop these links.

*“[My son]*, *he is going to have to be French*. *I don't know what position he will have in regard to his past*. *And therefore*, *as much as I don't want to hide it*, *I equally don't want to talk too much about it*. *I will try to see how he does with that and adapt to it*.” (Mother 2, of a 2-year-old)

*“If at a given point*, *she wants to learn about this culture*, *we will do it with her*.” (Father 12, of a 6-year-old)

#### Child's history before adoption

Seventeen parents in group 3 stressed their respect for their child's history. Parents in this group want to be able to give their children as much information as possible about their birth parents and their life before the adoption (photograph albums, videos, stories,…) and have searched (or are searching) actively for them. They think that one day their child will want to have this information.

*“I asked for lots of information about her parents*. *Not for myself*, *just to be able to answer the questions my daughter will have when she's older*.” (Mother 12, of a 6-year-old)

#### Contacts with other adoptive parents

In this group, 12 parents reported going online daily to internet forums (message boards) to discuss questions that they have about their children. All 12 were mothers. Questions about skin color, school problems, and parent-child relationships can be discussed. Associations of adoptive parents or internet groups play a role of support and enable experiences to be shared.

*“Adoptive parents support each other in France*, *communicate and share and give advice through Internet groups*.” (Mother 18, of an 8-year-old)

#### Travel to the country of birth

In group 3, 13 parents did not have, at the time of the study, plans to return to their child's country of birth. On the other hand, all of the parents in this group said that they would agree to accompany the child if he or she wanted to travel there. In 16 interviews, the parents nonetheless stressed the importance of time to think about it, that is, they would not immediately start the trip as soon as the child asked.

*“She said to me*: *I'd like to go to Haiti*. *To see my mother*. *Then I said to her*: *well*, *listen*, *for now you're too young*, *but I understand that you want to see your birth mother*, *and if you still want to go*, *we'll go*, *we'll arrange to go together*, *but now it's still a little early*.” (Mother 18, of a 8-year-old)

### Respondent validation

These results were sent by mail to 16 parents, who had agreed in advance to review the results. Ten of them responded. The themes uncovered by the qualitative analyses matched the ways the parents would have described the associations that they maintain with their child's country of birth and its culture. Of the 10 parents who responded, 2 had been classified in group 1, 3 in group 2, and 6 in group 3. Parents who responded validated the themes we found and agreed that they fit completely into one of the positions described (group 1, 2, or 3).

They emphasized the strength of their convictions about their position and their irritation at the discourse of French adoption agencies, which they find too rigid. They considered that they had developed their position about the child's cultural belonging very early, mainly before the child's arrival, and then a little at the time that they first met the child. Five parents again stressed the importance of the advice they received from parents' associations.

## Discussion

The qualitative phenomenological analysis of interviews with 51 adoptive parents shows three major groups of parental representations of the cultural identity of their internationally adopted child.

In group 1, which included 12 of the 51 parents (24%), parents deemed above all that their child had the same culture as they did and that maintaining associations, which they considered artificial, with the country of birth would prevent the child from feeling he or she belonged in their new family.

Parents in group 1 stated they need to “act as if” their children were French children born in France, to be able to make them a part of their family and so that they will not be seen as foreigners. They insist that there is no difference between an adopted child and a biological child. The parental position consisting of acting as if the child was born to them matches some parental positions described in the literature [47]. Parents in group 1 have a need to nullify the differences that exist between them and the child that they went to the other end of the world to find.

It is also akin to the first parental strategy described by Lee in his review of the literature on transracial adoptions: cultural assimilation, which includes parenting behaviors that reject differences or downplay the child’s unique racial and ethnic experiences [22,24]. This results in the child's acculturation into the majority culture [2]. A variant of cultural assimilation is a humanistic strategy emphasizing a “color-blind” orientation or a view of humanity without reference to ethnicity and race [2]. Several authors have described the color-blind approach to parenting [48,49]. Friedlander talks about a “universalist strategy" to describe the parental attitude that denies or minimizes the question of discrimination [28]. This attitude appears to be detrimental to the child [17].

This position, taken by the parents in group 1, also resembles that of some authors, especially European, who consider that making children members of the culture of the receiving country allows them to be assimilated and integrated completely both in their family and in the country of adoption [5,50].

In group 2 (18 parents of 51: 35%), on the contrary, the parents actively defended their children's bicultural identities. Through active links with the culture of the children’s country of birth, they allow them to develop competence in that culture. Studies demonstrate that children's level of cultural competence (for their culture of birth) depends on their parents' beliefs [6,7,8]. Parental cultural competence [7] is defined by active steps to promote the development of a positive cultural and ethnic identity in children as well as an awareness of the importance and role that ideas of ethnicity and culture occupy in each person's life. The consequence of this second point is parents' strong participation in helping their adopted children to develop the ability to protect themselves from racism and discrimination, even though the parents do not experience it themselves. Parents in group 2 therefore have a high level of parental cultural competence. The analysis of the interviews in group 2 shows these parents’ conviction that it is important to transmit to their children cultural elements of their country of birth, to facilitate their identity work. Pride in their cultural heritage enables better self-esteem.

In group 3, which contains 21 of the 51 parents (41%), adoptive parents have a fluid position that moves over time, determined by the child's questions. These parents think that children's feelings of belonging to their country of birth and its culture evolve with their age, their experience of discrimination, their questions about their identity, etc. This position resembles what Tessler, Gamache, and Liu describe as child choice, that is, adapting the connections with the country of birth and its culture according to the child's interest or choices [27].

This position follows the transnational and postcolonial approaches that reject a static, categorical, and reductive concept of cultural identity [51,52]. This criticism was initially formulated in the context of research about the identity building of immigrants and their children. These authors describe negotiations of identity and feelings of cultural belonging as subjective, complex, and dynamic [51]. Parents in group 3 also express their belief that their children's feelings of cultural belonging are unsettled and affirm that their role as parents is to adapt to their child's identity building process, regardless of its direction.

In all three groups, the interviews thus demonstrate the parents’ desire to protect the child. The parents in group 1 say they are protecting their children by assimilating them completely to the biological child they might have had; parents in group 2 say that they are protecting their children by actively developing their bicultural identity; and the parents of the group 3, by supporting and accompanying their children in the direction they choose, whether it is a refusal of or on the contrary a demand for associations with aspects of the culture of their country of birth.

Finally it is interesting to observe that the three parental positions identified in this study are consistent with and illustrate the different positions which appear to exist in the receiving countries. Our study must be seen in the historic and sociological context of France. These questions about the role of the birth country and its culture in adoptive families, which have considerably enriched the thinking about international adoptions in the United States for 15 years, have only been raised recently in France. We report here the first study of French adoptive parents about their representations and beliefs about the culture of their child's country of birth. The first reviews of the literature about the subject of the cultural identity of adopted children were only published in French journals in 2012 and 2014 [35,36]. The three groups found in this study are three faces of the debates in Europe and especially in France about the place of the culture of the birth country of internationally adopted children.

Another interesting descriptive point concerns the children’s characteristics in terms of age. The tables presented in the results call for three sets of comments.

- All three groups include recent adoptions (less than three years between adoption and interview) as well as later adoptions: 6 of 15 interviews involved recent adoptions in group 1, 6 of 24 in group 2, and 8 of 27 in group 3).
- Similarly, we find families that adopted a child at a young age (before the age of 1 year) in all three groups (5 of 15 interviews in group 1, 11 of 24 in group 2, and 6 of 27 in group 3).
- The parents' position does not seem to depend on the child's age at the time of the interview, either: 10 of 15 interviews involved children younger than 12 years at the time of the interview in group 1, 16 of 24 in group 2, and 19 of 27 in group 3. If we look more closely at the 23 parents whose children were 6 years or younger at the moment of their interview, 6 parents were in group 1, 7 in group 2, and 10 in group 3. Similarly, of the 10 parents with a child 3 years or younger at the time of the interview, 2 were in group 1, 4 in group 2, and 4 in group 3.

The parents' position about maintaining links with the child's country of birth and its culture therefore does not seem to be determined by the child's age at either the interview or at adoption or by the time that passed between the adoption and the interview. These results are supported by the results of the respondent validation, for the parents who responded said that they had considered this question about the child's country of birth before the child arrived. These convictions, which rest on educational values, seem to depend little on the children, their ages, or their characteristics.

## Limitations and Perspectives

One of the limitations of this study is the small number of fathers (13 fathers/38 mothers).

It would be interesting to describe the position of the children and adolescents themselves in relation to the question of their cultural belonging and compare them to their parents' positions. This will be the topic of a future study. The congruency between parent and adolescent perceptions of cultural activities and ethnic identity development has been explored by Scherman and Harre [26].

Finally it would be interesting to take into account the role of social welfare professionals who specialize in adoption. What advice have adoptive parents received from professionals about the connections to be maintained with the child's country of birth and its culture? In the current French context, which is evolving substantially on these questions, it is important to consider the discourse of professionals.

## Conclusion

Working with adoptive parents about their representations of their children's cultural belonging is an important issue. These representations, present from the very early stages of the adoption, determine the ways that the parents respond to their children when the latter ask the inevitable questions about their difference in physical appearance, their experiences of discrimination, their country of birth, and sometimes their desire to return there. It is also essential to explore these parental representations if we are to understand family interactions and provide more effective help to these adoptive families. Systematic training of adoption professionals around this question of parental representations of their child's cultural belonging must be set up in all countries receiving children in international adoptions, to enable early prevention, beginning with the child's arrival.

## Supporting Information

S1 Appendix Interview Protocol. (DOC)
