# Supplementary material for: Cultural Identity and Internationally Adopted Children: Qualitative Approach to Parental Representations
Source: PLoS One. 2015 Mar 16;10(3):e0119635. doi: 10.1371/journal.pone.0119635 (PMC4361487; doi:10.1371/journal.pone.0119635)
Supplement: S1 Appendix — (DOC) [file pone.0119635.s001.doc]

**Appendix S1: Interview Protocol**

In what country did you adopt your child?

Why did you choose to adopt in that country?

How old was the child when you adopted him/her?

Do you have any other children? If so, how many? What age are they? What are their names? and were they adopted?

Could you tell us why you chose to adopt?

What led to your child's being adopted? What do you know about your child's life before the adoption?

How did your child learn that s/he was adopted?

When did your child first become aware of the difference between you in physical appearance? How did s/he become aware of this?

Does your child ask you questions about his/her birth parents, and if so, what do you answer?

Is your child interested in his/her country of birth and does s/he ask you questions about it?

Are there things related to the country of birth that you do here in France?

Do you keep informed about the news in your child's country of birth?

Does your child speak or is s/he learning to speak the language of his/her country of birth? And how was this decision made?

Do you maintain contacts with your child's country of birth? If so, why and how? If not, why not?

Has your child already returned to his/her country of birth? And if so, how did that go, and if not, why not?

Do you talk to your child about the fact that s/he was born somewhere else? Are his/her cultural origins important for you?

After the adoption, has the question of physical appearance or skin color come up for you or your child?

Do you think that your child feels close to the culture of his/her country of birth?

Have you already talked about racism with your child?

Has your child already experienced racism? And if so, how did s/he react and how did you react?
